# Defining cell identity beyond the premise of differential gene expression

**DOI:** 10.1186/s13619-021-00083-7

**Published:** 2021-05-01

**Authors:** Hani Jieun Kim, Patrick P. L. Tam, Pengyi Yang

**Affiliations:** 1 School of Mathematics and Statistics, The University of Sydney, Sydney, NSW 2006 Australia; 2 Computational Systems Biology Group, Children’s Medical Research Institute, Faculty of Medicine and Health, The University of Sydney, Westmead, NSW 2145 Australia; 3 Charles Perkins Centre, The University of Sydney, Sydney, NSW 2006 Australia; 4 Embryology Unit, Children’s Medical Research Institute, Faculty of Medicine and Health, The University of Sydney, Westmead, NSW 2145 Australia; 5 School of Medical Science, Faculty of Faculty of Medicine and Health, The University of Sydney, Sydney, NSW 2006 Australia

## Abstract

Identifying genes that define cell identity is a requisite step for characterising cell types and cell states and predicting cell fate choices. By far, the most widely used approach for this task is based on differential expression (DE) of genes, whereby the shift of mean expression are used as the primary statistics for identifying gene transcripts that are specific to cell types and states. While DE-based methods are useful for pinpointing genes that discriminate cell types, their reliance on measuring difference in mean expression may not reflect the biological attributes of cell identity genes. Here, we highlight the quest for non-DE methods and provide an overview of these methods and their applications to identify genes that define cell identity and functionality.

## Main text

Defining the identity of a cell is fundamental to cell biology research (Kotliar et al. 2019; Morris 2019; Wagner et al. 2016; Weinreb et al. 2020). Traditionally, histological and morphological assessment of cells, overlaid with immunohistochemical information, has enabled us to identify cell types with confidence. Bulk RNA-sequencing (RNA-seq) preceded by FACS sorting has further unveiled the global molecular characteristics of cell populations of interest. However, these approaches have been restricted to cell types with known marker genes and the bulk measurement have masked the underlying cellular heterogeneity. Recent technological advances in genome-wide profiling of single cells have enabled the unbiased exploration of cell identity, allowing discovery of known and unknown cell types at single-cell resolution. Yet inferring the identity of cells has become a renewed challenge as the expanding breadth and depth of single-cell omics data now provide an unprecedented lens into the complexities and nuances of cellular identities.

For some cell types, the computational task to infer cell identity on the basis of omics profiles alone may be relatively straightforward, requiring the evaluation of the expression of known marker genes. For rare or previously unknown cell types, defining the gene set that uniquely identifies the cell is a challenge in the absence of any prior knowledge. This raises an important question of how we could select genes that mark a cell’s identity, henceforth referred to as cell identity genes (CIGs).

Many methods have been devised to identify CIGs, among which the most popular approach is based on differential expression (DE) of genes. A host of tools have been developed for DE analysis on bulk RNA-seq data, such as DESeq2 (Love et al. 2014), edgeR (Robinson et al. 2009), and Limma (Ritchie et al. 2015), and many of them have been successfully applied on single-cell data. Recent methods designed for mining single-cell gene expression data (Delmans and Hemberg 2016; Finak et al. 2015; Kharchenko et al. 2014; Pierson and Yau 2015; Qiu et al. 2017; Vallejos et al. 2015) address some confounding aspects of the analysis of scRNA-seq data, such as technical noise arising from variation in cellular detection rate, and attempt to capture more nuanced differences in cell-to-cell heterogeneity. However, whether these approaches faithfully capture CIGs remains unknown.

A common feature among most current DE methods is their reliance on a specific model of gene expression, which overlooks the heterogeneous nature of gene expression between cells and limits the discovery of CIGs by placing restrictions on the distribution of the genes selected. *t*-test based approaches (such as Limma) and MAST (Finak et al. 2015) assume a Gaussian distribution of gene expression; BASiCS, a Poisson distribution (Vallejos et al. 2015); and SCDE*,* a Poisson and negative binomial distribution (Kharchenko et al. 2014). As an illustration of the potential caveat, DE methods that are based on the Student’s *t*-test such as Limma (Ritchie et al. 2015) is that they prioritise genes that are stably expressed (i.e., conforms to a Gaussian distribution) in both the cell type of interest and other cell types as long as there are shifts in the mean expression. This means that any genes that do not follow this distribution are penalised irrespective of whether the gene may be critical to the identity of the cell or not, meaning that many marker genes identified by DE methods are simply more highly expressed in the cell type of interest than the rest.

**Fig. 1:**
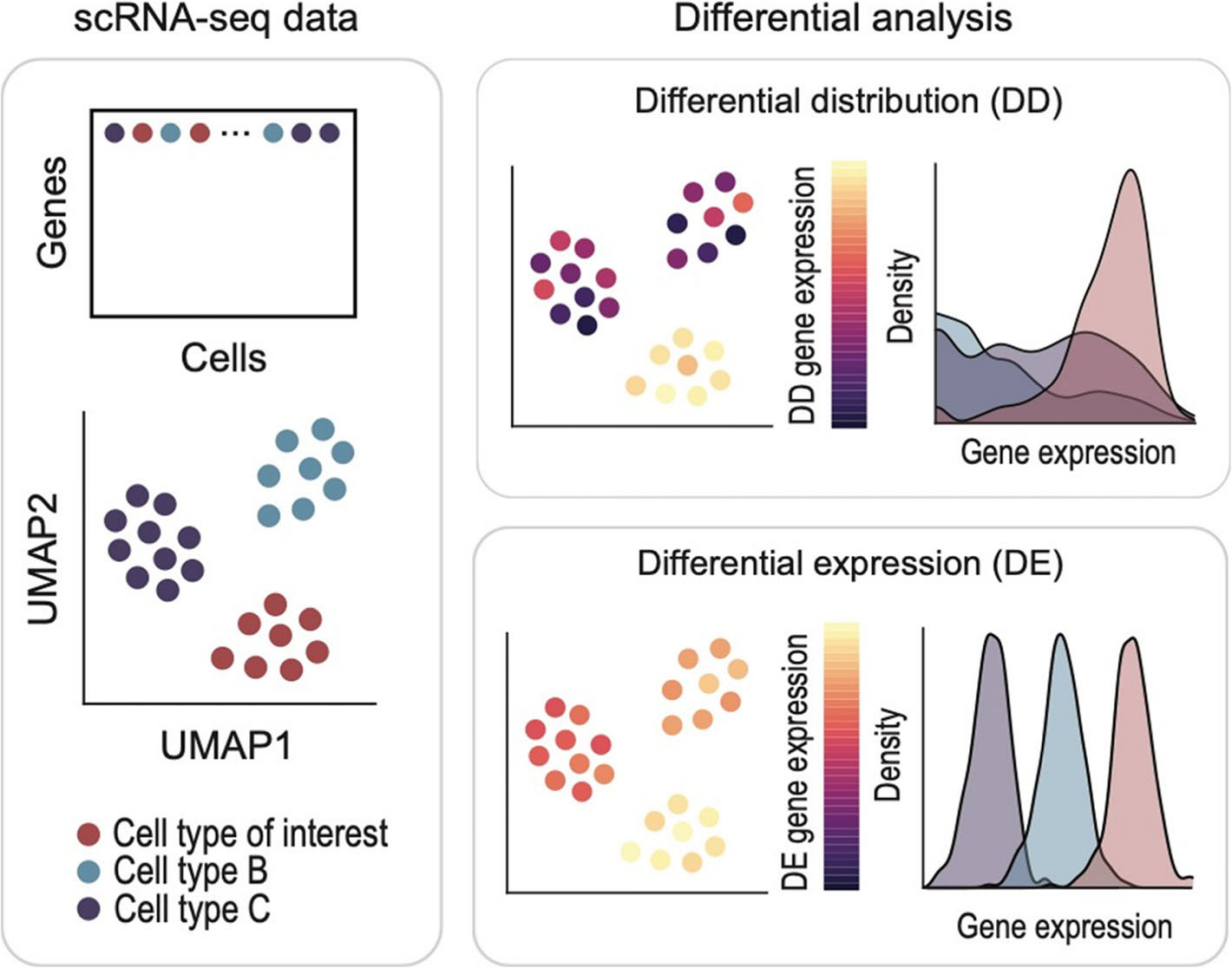
Schematic illustrating the expression of differentially distributed genes for a cell type of interest from single-cell RNA-seq data (scRNA-seq). The hypothetical scRNA-seq data consists of three cell types, which can be visualised as distinct clusters in the low dimensional space (left). Typical expression pattern of genes identified from using two differential analysis methods: differential distribution (top) and traditional differential expression (bottom)

Recently, methods based on new statistical metrics have been developed. These methods break away from detecting genes on the basis of shifts in means and attempt to capture more subtle differences in gene expression. For example, scDD is a blanket approach that detects differential proportion (DP), differential modes (DM), and bimodal distribution (BD), as well as DE (Korthauer et al. 2015). Non-parametric approaches are rarer, with model-free methods developed to find differential genes (Li and Tibshirani 2013; Tiberi et al. 2020). These metrics prioritise genes that are differentially distributed (DD) as opposed to those that are differentially expressed (Fig. 1). Whilst these methods are yet to be vetted in terms of their fidelity to prioritize genes that are robust in defining cell identities, they present new avenues for researchers go beyond finding genes that are most distinctively expressed in cells to those that may be more relevant to the identity of the cell and its phenotype.

A biological read-out that accurately captures cellular attributes would not only enhance our ability to assign cellular identities but also opens up a plethora of possibilities to investigate complex systems where assigning cellular identities is inherently more challenging. First, the availability of a comprehensive set of cell identity read-outs encompassing a wide range of cell types would enable a data-driven approach to accurately predict and quantitatively investigate new cell types. This kind of computational approach would not be limited to analysing new cell types but may be used to analyse how cell states are affected with disease or perturbations, capturing the nuance changes in the omics that would affect the overall phenotype of the cell. Second, assignment of cell identities of discrete cellular states, whilst of great importance, provides only a partial answer towards the greater goal of mapping all cellular states. Cells dynamically transition between discrete cell states or cell types, and this developmental landscape, as depicted in the Waddington’s model, illustrates the spectrum of states in which a cell may lie. Identifying the CIGs that define these transitional cell states will help us perform a much deeper analysis of cell identity characterisation and lineage differentiation.

In conclusion, with rapidly advancing single-cell technologies, the development of new computational methods that faithfully capture CIGs that are most relevant to the identity of cells is critical to advancing our knowledge of cellular identity. The selection of CIGs has major implications on a range of downstream single-cell computational applications, and oftentimes the biological interpretation hinges on the outcome of these downstream analyses. We aspire that enhancing our ability to identify CIGs will contribute towards and invigorate new research in elucidating the factors of cell identity and realising the potential of single-cell analytics technologies to pinpoint functional attributes that are relevant to the cellular phenotype.

## Abbreviations

scRNA-seq: Single-cell RNA sequencing
CIG: Cell identity gene
DD: Differential distribution
DE: Differential expression
DP: Differential proportion
DM: Differential modality

## References

[CR1] Delmans M, Hemberg M (2016). Discrete distributional differential expression (D3E) - a tool for gene expression analysis of single-cell RNA-seq data. BMC Bioinformatics.

[CR2] Finak G, McDavid A, Yajima M, Deng J, Gersuk V, Shalek AK, Slichter CK, Miller HW, McElrath MJ, Prlic M, Linsley PS, Gottardo R (2015). MAST: a flexible statistical framework for assessing transcriptional changes and characterizing heterogeneity in single-cell RNA sequencing data. Genome Biol.

[CR3] Kharchenko PV, Silberstein L, Scadden DT (2014). Bayesian approach to single-cell differential expression analysis. Nat Methods.

[CR4] Korthauer K, Chu L-F, Newton M, Li Y, Thomson J, Stewart R (2015). scDD: a statistical approach for identifying differential distributions in single-cell RNA-seq experiments. Genome Biol.

[CR5] Kotliar D, Veres A, Nagy MA, Tabrizi S, Hodis E, Melton DA, Sabeti PC (2019). Identifying gene expression programs of cell-type identity and cellular activity with single-cell RNA-Seq. Elife..

[CR6] Li J, Tibshirani R (2013). Finding consistent patterns: a nonparametric approach for identifying differential expression in RNA-Seq data. Stat Methods Med Res.

[CR7] Love MI, Huber W, Anders S (2014). Moderated estimation of fold change and dispersion for RNA-seq data with DESeq2. Genome Biol.

[CR8] Morris SA (2019). The evolving concept of cell identity in the single cell era. Dev.

[CR9] Pierson E, Yau C (2015). ZIFA: dimensionality reduction for zero-inflated single-cell gene expression analysis. Genome Biol.

[CR10] Qiu X, Hill A, Packer J, Lin D, Ma YA, Trapnell C (2017). Single-cell mRNA quantification and differential analysis with census. Nat Methods.

[CR11] Ritchie ME, Phipson B, Wu D, Hu Y, Law CW, Shi W, Smyth GK (2015). Limma powers differential expression analyses for RNA-sequencing and microarray studies. Nucleic Acids Res.

[CR12] Robinson MD, McCarthy DJ, Smyth GK. edgeR: a Bioconductor package for differential expression analysis of digital gene expression data. Bioinformatics. 2009;550:139–40. 10.1093/bioinformatics/btp616 PMC2796818 19910308

[CR13] Tiberi S, Crowell HL, Weber LM, Samartsidis P, Robinson MD. distinct:a novel approach to differential distribution analyses. bioRxiv. 2020; Available from. 10.1101/2020.11.24.394213.

[CR14] Vallejos CA, Marioni JC, Richardson S (2015). BASiCS: Bayesian analysis of single-cell sequencing data. PLoS Comput Biol.

[CR15] Wagner A, Regev A, Yosef N (2016). Revealing the vectors of cellular identity with single-cell genomics. Nat Biotechnol.

[CR16] Weinreb C, Rodriguez-Fraticelli A, Camargo FD, Klein AM (2020). Lineage tracing on transcriptional landscapes links state to fate during differentiation. Science.

